# High Expression of Complement Components in the Kidneys of Type 2 Diabetic Rats With Diabetic Nephropathy

**DOI:** 10.3389/fendo.2019.00459

**Published:** 2019-07-09

**Authors:** Yinqiong Huang, Jinting Xu, Xiaohong Wu, Xiaoyu Chen, Xuefeng Bai, Yong Zhuang, Jingwen Fang, Xiahong Lin

**Affiliations:** ^1^Department of Endocrinology, The Second Affiliated Hospital of Fujian Medical University, Quanzhou, China; ^2^Department of Endocrinology, Jinjiang Municipal Hospital, Jinjiang, China

**Keywords:** type 2 diabetes, diabetic nephropathy, complement components, mannose-binding lectin pathway, renal tubule

## Abstract

**Background:** Diabetic nephropathy (DN) is the leading cause of end-stage failure of the kidneys; however, its pathogenesis remains unknown. This study assessed the expression of complement components in the kidneys of rats with type 2 DN to investigate their role in DN.

**Methods:** A rat model of type 2 DN was induced by a high-fat diet combined with low-dose streptozotocin. Blood glucose, fasting insulin levels, insulin resistance index, and 24-h urinary albumin excretion (UAE) were measured. Renal tissue morphological features were observed. The mesangial index and arteriosclerosis index were calculated. Immunohistochemistry and western blot were used to measure the expression of complement components in the kidneys.

**Results:** The kidney weight: body weight (mg/g) ratio in the DN group was significantly greater than those in the control and diabetes mellitus (DM) groups. The arteriosclerosis index, mesangial index, and tube area percentage in the DN group were significantly higher than those in the control and DM groups, but these parameters did not significantly differ between the control and DM groups. The expression of the complement components C1q, mannose-binding lectin (MBL), mannan-binding lectin-associated serine protease (MASP)-2, B factor, C3, and C5b-9 in the DN group was significantly higher than that in the control and DM groups but did not significantly differ between the control and DM groups. Most of the complement components were mainly expressed at the renal tubular site. Correlation analysis showed that 24-h UAE were positively correlated with C1q, MBL, MASP-2, B factor, and C5b-9 expression. MI was positively correlated with MBL, B factor, C3, and C5b-9 expression. AI was positively correlated with C1q, MBL, MASP-2, and B factor expression.

**Conclusion:** Complement components including C1q, MBL, MASP-2, B factor, C3, and C5b-9, were highly expressed in the kidneys of type 2 diabetic rats with DN. Most of the complement components were mainly expressed in the renal tubules. High expression of complement components was found to be associated with the progress of DN. Our study suggests that complement system activation is a progressive factor in type 2 diabetic nephropathy. Inhibition of pathological complement activation may be a promising therapeutic strategy for DN.

## Introduction

Diabetes mellitus (DM) has become a global public health problem, and its prevalence is increasing worldwide (1). Diabetic nephropathy (DN) is the most common cause of chronic kidney disease, accounting for ~50% of cases in the developed countries (2). In developed countries, DN is the primary cause of end-stage renal disease (3). However, the pathogenesis of DN remains unclear. Advanced glycation end products produced because of long-term hyperglycemia, activation of protein kinase C, enhanced expression of transforming factor β, and oxidative stress are the main causes of DN (4). An increasing number of studies suggest that activation of the complement system is involved in the pathogenesis of DN. Renal glomerular complement C3 deposition has been observed in mice with type 1 diabetes mellitus (TIDM) and mice with T2DM (5, 6). Glomerular deposition of the membrane attack complex (MAC) C5b-9 has been detected in patients with diabetes mellitus (DM) (7, 8). C3a-mediated pre-inflammatory and pre-fibrotic responses in rats with T2DM aggravate renal damage (9), while inhibition of complement C5 could attenuate glomerular mesangial proliferation and urinary protein excretion in rats (10), suggesting that complement system activation is associated with the pathogenesis of DN.

Activation of the complement system includes three pathways: the classical pathway, lectin pathway, and alternative pathway. Their common product is C3 convertase, which activates the downstream complement response to form the MAC (11). In recent years, studies have indicated that the lectin pathway might be involved in the pathogenesis of DN. In the lectin pathway, mannose-binding lectin (MBL) and ficolin recognize and bind mannose, fucose, and *N*-acetyl glucose on the surface of pathogenic microorganisms; then, they activate mannan-binding lectin-associated serine proteases (MASPs), mainly MASP-2, to activate downstream components of the complement system (12, 13). Normally, MBL and ficolin do not bind to their own tissues. However, in diabetes, glycosylation induces such interactions of MBL and ficolin, which leads to systemic and local inflammatory reactions and eventually, diabetic complications (14, 15).

Previous studies have shown MBL deposition in the glomeruli of mice with T1DM (16–18). MBL-knockout mice with T1DM showed a significant decrease in kidney weight, urinary albumin excretion (UAE) rate, and type IV collagen expression (19). Serum MBL levels significantly increase in patients with T2DM nephropathy, and MBL levels have been found to be predictive of the risk of progression to DN (20, 21). MASP-2 is a key molecule in the activation of the lectin pathway. However, whether MASP-2 is involved in the pathogenesis of DN is not known. Only one study has been performed on this topic; it was found that the MASP-1 and MASP-2 serum levels in patients with T1DM were significantly higher than those in the control group (22). Synthesis of complement factors mainly occurs in the liver, while activation and cascade initiation occur in the blood circulation. However, to our knowledge, no study has been performed on whether complement components are highly expressed in the kidneys in DN (23). Therefore, this study aimed to assess the expression of complement components in the kidneys of rats with type 2 DN to investigate their role in DN.

## Materials and Methods

### Laboratory Animals and Reagents

Twenty-four 5-week-old male Sprague Dawley (SD) rats (weight, ~150 g) were purchased from the Animal Experimental Center of Fujian Medical University. All rats were housed under standard conditions (constant temperature, constant humidity conditions, and a 12-h light/dark cycle), with free access to food and water. The study followed the National Guidelines for Laboratory Animal Welfare and was approved by the Experimental Animal Ethics Committee of Fujian Medical University (SYXK 2016-0006FJMU IACUC 2016-024).

Streptozotocin (STZ) was purchased from Sigma Chemicals USA. Enzyme-linked immunosorbent assay (ELISA) kits for serum insulin and urinary albumin were purchased from Shanghai Xitang Company. Complement protein primary antibody was purchased from England Abcam, the secondary antibody was purchased from Beijing Emarbio Science & Technology Company.

### Establishment of a Rat Model

The 24 rats were acclimatized for 1 week before conducting the experiments. Then, they were randomized to three groups: control group (*n* = 8), type 2 diabetes group (DM group, *n* = 8), and type 2 DN group (DN group, *n* = 8). The control group was fed a normal diet (Research Diets, D12450h), and the DM group and DN group were fed a high-fat diet (Research Diets, D12451) for 8 weeks. Subsequently, fasting insulin (FINS) and fasting glucose were measured to calculate the homeostasis model assessment of insulin resistance (HOMA-IR = FINS × FPG/22.5). Then, the DM group and DN group were injected intraperitoneally (i.p.) with STZ at a low dose of 30 mg/kg diluted in citrate buffer, while the control group was injected with citrate buffer. Diabetes was confirmed by measuring glucose levels after 72 h of STZ injection. Animals showing a glucose level of ≥16.7 mmol/L for 3 consecutive days were considered to be diabetic (24). The DN group continued to be fed the high-fat diet for 8 weeks to establish a DN model. Rats were housed in separate metabolic cages; 24-h urine was collected and UAE was measured. The DN model was confirmed on obtaining 24-h UAE ≥ 30 mg/24 h (25).

### Biochemical Indicator Test

Fasting insulin levels and UAE were measured with an insulin ELISA kit and a urinary albumin ELISA kit following the reagent's instructions.

### Renal Pathology

The animals were anesthetized by intra-peritoneal injection of ketamine/xylazine. All efforts were made to minimize the suffering of the animals. Then their kidneys were removed and weighed. After the kidneys were fixed in 4% paraformaldehyde and embedded in paraffin, the kidney tissues were cut into 4-μm-thick sections and stained using hematoxylin-eosin (HE) and periodic acid–Schiff (PAS) staining. Then, the mesangial index (MI; MI = mesangial matrix area/glomerular area × 100%), arteriosclerosis index (AI; AI = wall area/ [wall area + lumen area] × 100%), and tube area percentage (TAP; TAP = tube area/total field area × 100%) were calculated.

### Immunohistochemical Analysis of Renal Complement Components

The kidneys were fixed in 4% paraformaldehyde solution, embedded in paraffin, and cut into 3-μm-thick sections. After dewaxing and tissue antigen repair, goat serum in concentration of 10% was added and the sections were incubated for 1 h at room temperature (23–27°C). Phosphate-buffered saline (PBS) was added to the controls and primary antibody diluted by 10% goat serum was added to the remaining samples; the sections were incubated at 4°C overnight and then rewarmed at 37°C for 20 min. Subsequently, the sections were washed three times with PBS and secondary antibody was added, following which they were incubated at 37°C for 30 min, washed three times with PBS, and developed with DAB. Then hematoxylin counterstaining, dehydration, transparency, sealing, and microscopic examination were done. Brown coloration obtained during immunohistochemical analysis indicated positive results. Five fields of view were randomly selected under 400-fold magnification per slice. The integrated optical density (IOD) for positive results was analyzed using the ImageJ software (Wayne Rasband National institutes of Health, USA), and the results were subjected to statistical analysis.

### Western Blot Analysis of Renal Complement Components

The kidney tissues were lysed and total protein was extracted. Then, 30 μg protein samples were separated using sodium dodecyl sulfate (SDS)/polyacrylamide gel electrophoresis (PAGE), transferred to a polyvinylidene difluoride (PVDF) membrane, and blocked with milk/TPBS (Tris-Buffered Saline Tween-20) blocking solution for 2 h at room temperature. Primary antibody was added to the PVDF membrane (1:1,000 dilution), and the membrane was incubated overnight at 4°C. Horseradish peroxidase–labeled secondary antibody (1:10,000 dilution) was added the next day for 2 h at room temperature. The blot was developed with an ECL kit, exposed to a gel imaging system, and analyzed using the Image Lab software.

### Statistical Analyses

All statistical analyses were performed using the SPSS Statistics 20 software. Data have been expressed in terms of mean ± standard deviation. Normally distributed data were analyzed using one-way analysis of variance (ANOVA), and non-normal distributions were analyzed with the Kruskal–Wallis H test. Normally distributed data were analyzed with the Pearson test for related variables, and non-normally distributed data were analyzed with the Spearman test. Statistical differences were considered as significant if the *P* < 0.05.

## Results

### Establishing of T2DM Rats With DN

**Figure 1:**
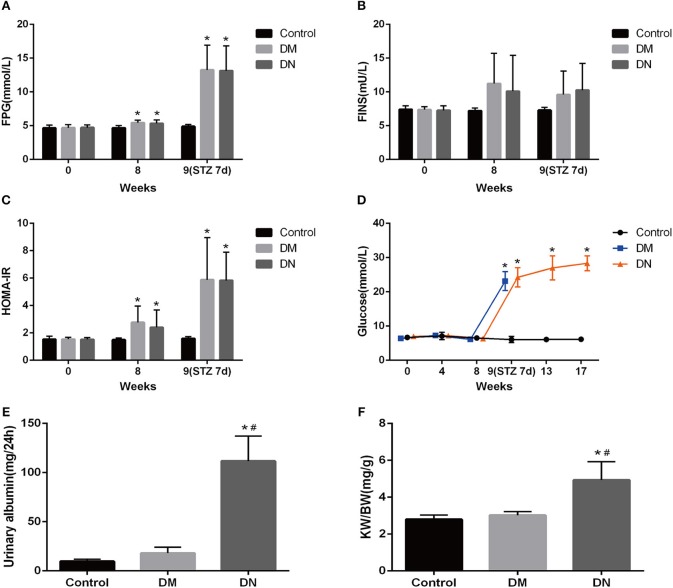
Biochemical indicators in the modeling process of type 2 diabetic nephropathy. **(A–C)** Fasting blood glucose **(A)**, fasting insulin **(B)**, and insulin resistance index **(C)** during the modeling process. **(D)** Blood glucose of rats in each group during modeling. **(E)** 24-h UAE in each group of rats. **(F)** Kidney weight: body weight ratio of each group. Data are presented as mean ± SD. *n* = 8. *P < 0.05 vs. Control, ^#^*P* < 0.05 vs. DM group.

After 8 weeks of high-fat diet, the HOMA-IR in the DM group and DN group was significantly higher than that in the control group (Figures 1A–C), suggesting that insulin resistance occurred in the DM group and DN group. STZ injection resulted in significant increase in glucose levels in the DM group and DN group as compared to those in the control group (Figure 1D), suggesting that the T2DM model was successfully established. The UAE in the DN group was higher than that in the control group and the DM group (Figure 1E). The kidney weight: body weight ratio (KW/BW) of the DN group was significantly higher than that of the control group and the DM group (Figure 1F).

### HE Staining

**Figure 2:**
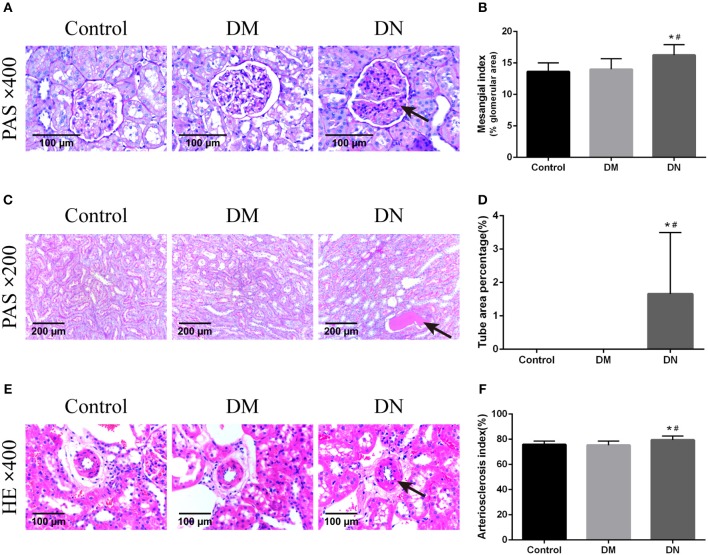
Pathological morphological features of kidney tissues in each group of rats. **(A)** Pathological morphological features of glomeruli in each group of rats. The arrow shows mesangial hyperplasia. **(B)** Mesangial index of rats in each group. **(C)** Pathological morphological features of renal tubules in each group. The arrow shows the protein-like cast. **(D)** Tube area percentage of each group of rats. **(E)** Pathological morphological features of renal arterioles in each group. The arrow shows thickening of the arteriolar wall. **(F)** Renal arteriosclerosis index of rats in each group. Data are presented as mean ± SD. *n* = 8. *P < 0.05 vs. Control, #*P* < 0.05 vs. DM group.

HE staining was used to assess the glomerular volume, glomerular capillary loops, mesangial area, renal tubular epithelial cells, and renal interstitium. Compared with the control group and DM group, the major pathological changes in the DN group were glomerular hypertrophy, mesangial cell proliferation, lobulated capillary, and Bowman's capsule narrowing; furthermore, the wall of the interlobular artery and the basement membrane of the renal tubule showed thickening, the epithelial cells were swollen, and protein-like casts were noted. However, the morphological features of kidney tissues did not significantly differ between the DM group and control group. The MI (26) of the DN group was higher than that of the control group and DM group (Figures 2A,B). The TAP for the DN group was higher than that for the control group and DM group (Figures 2C,D). The AI was higher in the DN group than in the control group and DM group (Figures 2E,F). The indexes including MI, TAP and AI did not significantly differ between the control group and DM group (*P* > 0.05).

### Immunohistochemical Method to Detect the Expression of Complement Components in the Kidneys

**Figure 3:**
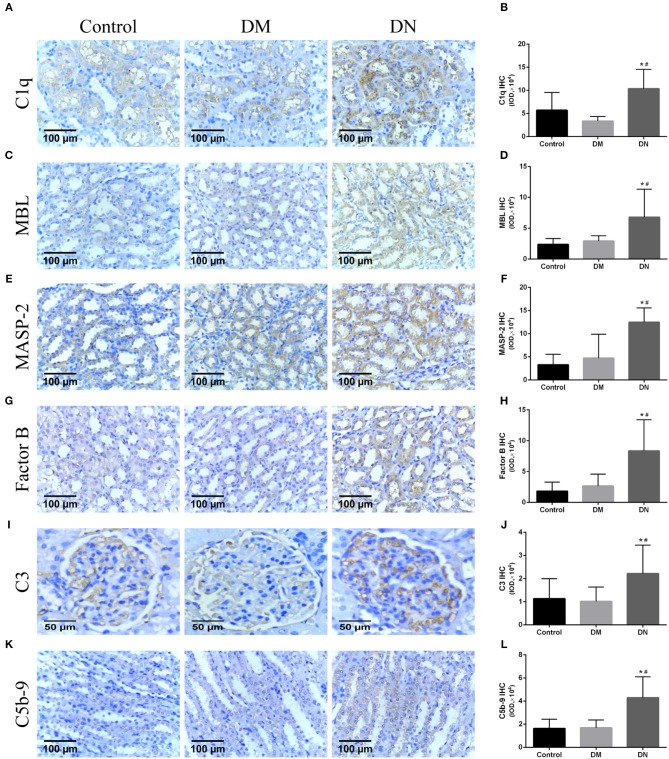
Immunohistochemical results for complement components expression in rat kidneys (27). **(A,B)** C1q expression, **(C,D)** MBL expression, **(E,F)** MASP-2 expression, **(G,H)** B factor expression, **(I,J)** C3 expression; **(K,L)** C5b-9 expression. Data are presented as mean ± SD. *n* = 8. *P< 0.05 vs. Control, #*P* < 0.05 vs. DM group.

Positive expression of immunohistochemical by IOD (× 10^4^) showed that the expression level of the renal complement C1q in the DN group was higher than that in the control group and DM group (Figures 3A,B). The MBL expression level in the DN group was higher than that in the control group and DM group (Figures 3C,D). The MASP-2 expression level in the DN group was higher than that in the control group and DM group (Figures 3E,F). The factor B expression level in the DN group was higher than that in the control group and DM group (Figures 3G,H). The C3 expression level in the DN group was higher than that in the control group and DM group (Figures 3I,J). The C5b-9 expression level in the DN group was higher than that in the control group and DM group (Figures 3K,L). Moreover, most of the complement components, including C1q, MBL, MASP-2, B factor, and C5b-9, were mainly expressed in the renal tubular cells, and C3 was mainly expressed in the glomerulus.

### Western Blotting to Detect the Expression of Complement Components in the Kidneys

**Figure 4:**
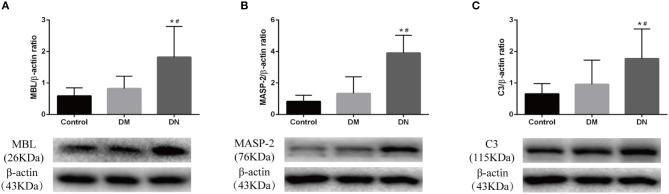
Western blotting results for complement component expression in rat kidneys. **(A)** MBL expression; **(B)** MASP-2 expression; **(C)** C3 expression. The molecular weight of beta-actin, Masp-2 and C3 is 43, 76, and 115 kDa, respectively. We analyzed the three proteins on the same gel, that is, Masp-2 and C3 were compared to beta-actin on the same gel. Each column represents the mean value that was derived from eight mice in each group, that was eight lanes per experimental group. Data are presented as mean ± SD. *n* = 8. *P < 0.05 vs. Control, ^#^*P* < 0.05 vs. DM group.

Western blotting was used to detect the expression of complement components in the kidneys. The relative gray value showed that MBL expression in the DN group was higher than that in the control group and DM group (Figure 4A). MASP-2 expression in the DN group was higher than that in the control group and DM group (Figure 4B). C3 expression in the DN group was higher than that in the control group and DM group (Figure 4C). The expression of complement components did not significantly differ between the control group and DM group.

### Correlation Analysis Between Complement Levels and Kidney Parameters

As shown in Table 1, 24-h UAE was positively correlated with C1q, MBL, MASP-2, B factor, and C5b-9. MI was positively correlated with MBL, B factor, C3, and C5b-9. AI was positively correlated with C1q, MBL, MASP-2, and B factor.

**Table 1:** Correlation analysis of complement components and kidney parameters.

	**24-h UAE**		**MI**		**AI**	
	***r***	***p*-value**	***r***	***p*-value**	***r***	***p*-value**
C1q	0.580	0.003	0.368	0.076	0.481	0.017
MBL	0.640	0.001	0.457	0.025	0.465	0.022
MASP-2	0.751	<0.001	0.352	0.091	0.494	0.014
B factor	0.728	<0.001	0.429	0.036	0.486	0.016
C3	0.346	0.098	0.410	0.046	0.308	0.143
C5b-9	0.718	<0.001	0.498	0.013	0.393	0.057

## Discussion

To date, the pathogenesis of DN remains unclear. Mounting evidence has indicated that activation of the complement system is involved in DN. In the Zucker rat model of T2DM, the C3, C4, C5, C6, C8, and C9 levels were found to be elevated after ischemia (28). Serum and renal complement C3 levels were found to increase in diabetic patients with kidney disease (29, 30). Increased MBL levels were reported to be related to increased risk of DN and significantly increased risk of death in patients with T1DM (31–33). To our knowledge, no studies have reported the role of MASP-2, the key molecule in the lectin pathway, in type 2 DN. In addition, it is unclear whether the expression of complement components in the kidneys increases in DN.

Complement molecules are mainly produced by hepatocytes, and studies have shown that extrahepatic tissues including kidney, brain, blood vessels, lungs, and intestines can synthesize a small amount of complement components, of which the kidney is one of the main sites for the synthesis of complement components outside the liver (34). Endothelial cells, epithelial cells, and tubular cells all have the ability to synthesize complement molecules (35). Expression of complement in kidney is associated with many kidney diseases. Local synthesis of complements, including C1q, C1r, C1s, and C3, increases in the entire kidney during kidney fibrosis (36). The current study analyzed the expression of complement components in the kidneys of rats with type 2 DN. It was found that the expression levels of most of the complement components, including C1q, MBL, MASP-2, B factor, C3, and C5b-9, increased in rat kidney tissues in DN. In addition, they were related with 24-h UAE, MI, and AI levels. The expression of complement components in the kidneys increased significantly in DN, but not in DM, which indicated that the complement system aggravated the progress of DN rather than initiating the progress.

Glomerular damage is generally considered to be the main cause of microalbuminuria and early kidney damage (37). It has been reported that the immunological activity of apoptotic proteins such as Bax and caspase-3 are increased in the glomerulus of the untreated diabetic group (38). Significant deposition of lipid peroxidation biomarkers was found in the glomerulus in T2DM rats (39). As well-some studies suggested that tubular damage was the main cause of early renal disease. Albuminuria occurs before glomerular lesions and urinary albumin is a sensitive indicator of early tubular damage (40). In the proximal tubule cells, the increased absorption of renal protein can lead to tubular damage by the cytokines and chemokines, which can enhance the inflammatory response and activate the interstitial fibrosis process (41). Activation of the complement system has been found in patients with diabetic nephropathy. C1q, C4d, and C5b-9 deposits in glomerular were more prevalent in cases with DN than in cases without DN, and glomerular C4d and C5b-9 deposits were correlated with the severity of DN (42). Another study showed immunostaining for C1q, C3c, C4c,C5, C9, and factor H were found in the kidney cortex and medulla of sheep with acute kidney injury (43). In the current study, most of the complement components were mainly expressed in the renal tubules, which suggests that activation of the complement system plays an important role in tubular injury than glomerular damage in DN.

To conclude, our study showed that the expression of complement components in rat kidneys itself increased in type 2 DN and was associated with the progress of DN. The study suggests that the activation of the complement system plays a role in the progression of type 2 diabetic nephropathy and provides an insight into the mechanism of diabetes. Thus, inhibition of pathological complement activation may be a promising therapeutic strategy for DN, as well as for some other disorders such as atypical hemolytic uremic syndrome, allergy, infection, which are related to the over activation of the complement system (44, 45).

This study has some limitations. The circulating complement components were not measured because of inadequate volume of serum samples. The evidence for a causal relationship between the complement system and progress of DN is insufficient and further research is required to confirm it. In future studies, we will use an appropriate complement inhibitor such as the C5 inhibitor eculizumab (46), anti-MASP-2 monoclonal antibody (AbD04211) or complement knock out model to further explore the difference in renal disease progression between diabetic rats after complement inhibition and uninhibited rats, so as to clarify the causal relationship between complement or one of the pathways and the occurrence and progression of diabetic nephropathy. We predict that diabetic rats that inhibit complement system activation will show reduced kidney disease.

## Ethics Statement

The study followed the National Guidelines for Laboratory Animal Welfare and was approved by the Experimental Animal Ethics Committee of Fujian Medical University (SYXK 2016-0006FJMU IACUC 2016-024).

## Author Contributions

YH, JX, and XW contributed to the study equally. YH, JX, XW, and XL conceptualized and designed these studies, performed them, and wrote the manuscript. XC, XB, YZ, and JF contributed through data analyses, data interpretation, and manuscript preparation. All authors contributed to manuscript revision and read and approved the submitted version.

### Conflict of Interest Statement

The authors declare that the research was conducted in the absence of any commercial or financial relationships that could be construed as a potential conflict of interest.
